# Immunosuppression and apoptosis activation mediated by p53-Bcl2/Bax signaling pathway -The potential mechanism of goldfish (*Carassius auratus Linnaeus*) gill disease caused by *Myxobolus ampullicapsulatus*

**DOI:** 10.3389/fimmu.2022.998975

**Published:** 2022-08-30

**Authors:** Senyue Liu, Lin Luo, Fengyuan Zuo, Yi Geng, Yangping Ou, Defang Chen, Shiyong Yang, Wei Luo, Yan Wang, Jun Wang, Xiaoli Huang

**Affiliations:** ^1^ Department of Aquaculture, College of Animal Science & Technology, Sichuan Agricultural University, Chengdu, China; ^2^ Department of Basic Veterinary, College of Veterinary Medicine, Sichuan Agricultural University, Chengdu, China; ^3^ Department of Aquaculture, College of Life Science, Neijiang Normal University, Neijiang, China

**Keywords:** *Myxobolus*, p53-Bcl2/Bax signaling pathway, apoptosis, immunosuppression, gill

## Abstract

*Myxobolus*, a major harmful type of *myxospora*, is one of the main parasitic pathogens of freshwater fish. Once myxoboliosis occurs, treatment can be extremely difficult. Therefore, clear understandings of the etiology of myxoboliosis and its pathological mechanism are keys for prevention and control. Here, histology, transmission electron microscopy, transcriptome study, tunel assay, and immunohistochemistry were carried out, revealing the morphology, pathological effects as well as host response mechanism of goldfish gill to *Myxobolus ampullicapsulatus*. Histological studies showed that the mature spores of *Myxobolus ampullicapsulatus* were composed of three parts, the spore shell, sporoplasm and bottle shaped polar capsule containing double S-shaped polar filaments. Transcriptome analysis revealed that *Myxobolus ampullicapsulatus* -infected (Myx) goldfish gills were characterized by apoptosis activation mediated by “p53 signaling pathway” with significantly up-regulated apoptosis-related differential genes dominated by p53-Bcl2/Bax signaling pathway. In addition, tunel assay revealed severe gill apoptosis in the Myx group. Transcriptome analysis also revealed that Myx group showed changes in immune response and significantly down-regulated immune-related differential genes. Beyond that, immunohistochemistry showed that there was no significant increase in the number of gill lymphocyte after parasite infection. These results suggest that the pathological mechanism of *Myxobolus ampullicapsulatus* infection on gills of goldfish may be related to apoptosis and immunosuppression. Subsequent qRT-PCR showed that apoptosis-related genes (*Caspase3,Bad, Bax*) and anti-inflammatory gene *IL-10* were significantly increased, while immune-related pro-inflammatory genes (*IL-1β, IL-8*) were markedly down-regulated, further verifying the transcriptome results. Based on the above results, we concluded that p53-Bcl2/Bax related networks that dominant the expression of apoptosis genes were activated while immunity was suppressed in the gills of *Myxobolus ampullicapsulatus* infected goldfish. Our study is not only of benefit to enrich the taxonomy of *Myxobolus* but also clarifies its pathogenic mechanism, thus providing targets for prevention and control of myxoboliosis.

## Introduction

*Myxosporean*, a common metazoan parasite, is widely dispersed around the world and primarily parasitizes fish. Currently, more than 2500 *Myxosporean* species have been recorded worldwide (1), causing physiological abnormalities, as well as affecting the growth and development of the host. In addition to causing multiple fish diseases, *myxosporean* infection can directly lead to the death of the host, resulting in serious economic losses and ecological pollution (2). *Myxobolus*, the most abundant species of *myxosporean*, is one of the most harmful *myxospora* groups to fish (2). As a major aquaculture nation in the world, China has been impacted by the disease of *myxobolus* for a long time.

From March to October, *myxobolus* disease, which parasitic on the body surface, gills, pharynx, liver, intestine, oral cavity and other tissues of fish, often occurs (3, 4). Diverse types of *Myxobolus* parasitize different host and cause distinct pathogenic alterations. *Myxobolus honghuensis* mainly parasitized on the pharynx of *Carassius auratus*, resulting in the swelling of the host pharynx and the lifting of the gill cover, which ultimately caused the afflicted fish to have trouble breathing and eating (5, 6). *Myxobolus wuli* mainly parasitized in *Carassius auratus gibelio* liver, forming a huge cyst in liver, causing abdominal bulge of the host (6, 7). *Myxobolus ampullicapsulatus* mainly parasitize the gills of *Carassius auratus*, forming white cysts with a circular shape of 1.2 to 3mm which contain abundant mature spores with two bottle shaped polar capsule, resulting in hyperemia, swelling and degeneration of gill filaments. (8, 9). Therefore, to prevent and control the occurrence of such diseases in aquaculture, it is necessary to comprehend the pathogen of *myxobolus* and investigate its pathogenic process.

*Myxobolus* parasitizes fish tissues, and mature spore has a tough sporocyst shell, which is changeling for drug therapy to penetrate into (10, 11). Once spores released into the water from diseased fish, are ingested by fish or enter the body surface through wounds, the spores will continue to multiply in the fish, leading to widespread outbreaks. At present, the pathogenic process of myxoboliosis is vague, and there is no appropriate targeted therapeutic therapy (12). As a consequence, it is vital to investigate the pathogenic mechanism of *myxobolus* to fish and prevent the disease from the perspective of the host.

Goldfish, *Carassius auratus Linnaeus* (Cypriniformes, Cyprinidae, Carassius), is one of the most popular ornamental fish around the world. A total of 392,507 million freshwater ornamental fish were bred in China in 2019, and the export trade value was as high as $4,201,600 (13). However, after the *Myxobolus* parasitizes goldfish, spore frequently form white comedoid cysts that accumulate into nodules, causing hyperemia or ulceration of host tissue (14), which greatly affects the ornamental value of goldfish and results in a mortality rate of up to 70%, causing significant economic losses. Thus in view of the economic value, sensitivity to *Myxobolus*, ease access to goldfish, this study extensively collected goldfish infected with *Myxobolus*, and used histology, molecular biology, transcriptome as well as other methods to classify and identify *Myxobolus*, and explored its infection mechanism to goldfish gills, which enriched the morphological characteristics of *Myxobolus*, deepened the understanding of the pathogenesis of myxoboliosis, and was conducive to the formulation of targeted prevention and control plans.

## Materials and methods

### Experimental fish and tissue sampling

30 goldfish (6.55 ± 0.18 g) with *Myxobolus ampullicapsulatus* (Myx group for short) and 30 goldfish (6.28 ± 0.34 g) without parasites infection (control group for short) used in this study were obtained from fish farms in Chengdu (Sichuan province, China) through multiple collections. After each collection, anesthetized goldfish with MS222 (Aladdin, China), immediately collected gill tissue samples for transmission electron microscopy observation, Hematoxylin and eosin (H&E) stain, TUNEL assay, immunohistochemistry and RNA extraction as described below. Animal procedures were approved by the Animal Experiment Committee of Sichuan Agricultural University and carried out according to the relevant guidelines.

### DNA extraction, amplification, and sequencing

DNA (gDNA) from gills of three goldfish with *Myxobolus ampullicapsulatus* were extracted using DNeasy^®^ Tissue&Blood Kits (Qiagen, Hilden, Germany) according to the manufacturer’s instructions. Specific primers of *Myxobolus* (5′- TTC TGC CCT ATC AAC TTG TTG-3′ and 5′- CTA CGG AAA CCT TGT TAC G-3′) were set for PCR amplification. PCR amplifications were performed using a ProFlex PCR System (Thermo Fisher Scientific Inc., Waltham, Massachusetts, USA) with total volume of 25 μL containing 12.5 μL dNTP mix, 2 μl of gDNA, 1 μL of the forward and reverse primers and 8.5 μL of double distilled water. The reaction conditions used were as follows: 94°C for 3 min, followed by 32 cycles of 94°C for 10 s, 60°C for 10 s and 72°C for 10 s and elongation at 72°C for 5 min. PCR products were then pooled and sent to a commercial company (Tsingke Biotechnology, China) for purification and sequencing (Sanger). Sequences were edited using the software Geneious (Saint Joseph, Missouri, USA) to check electropherograms.

### Sequence alignment and phylogenetic analysis

Nucleotide BLAST analysis of amplified sequences obtained in this study was performed on the website of the National Center for Biotechnology Information (http://www.ncbi.nlm.nih.gov/blast). Clustal W software was used for multi-sequence alignment, MEGA 10.0 software was used to construct the phylogenetic tree by neighbor-joining method with 1,000 non-parametric bootstrap replicates.

### Transmission electron microscopy

The *Myxobolus ampullicapsulatus* infected gill was fixed with 2.5% glutaraldehyde at 4°C for 24h, washed with PBS (pH 7.2), fixed with 1% osmium acid, rinsed with PBS, dehydrated with serial acetone, penetrated, embedded, ultrathin section, double stained with uranium acetate and lead citrate. Micrographs were taken with transmission electron microscopy (TEM, Hitachi H-7500, Japan) operating at 80 kV.

### Total RNA extraction and cDNA synthesis

Twelve goldfish (6 samples per group) were randomly selected and the second gill on the left side was collected, from which RNA was extracted using TRIzol reagent (Invitrogen, USA) following the manufacturer’s guideline. Subsequently, the concentration and purity, the integrity of RNA, as well as the RIN value were evaluated with Nanodrop 2000 spectrophotometer (Thermo Scientific, USA), a 1.2% (w/v) agarose gel electrophoresis and Agilent 2100, respectively. An equal amount of total RNA (1µg) was incubated with RNase-Free ddH_2_0 and gDNase Mix to eliminate contaminated gDNA and normalize gene expression levels for each sample. Then reverse transcription was performed in a 20 μL reaction volume containing 10 μL of the template above, 4 μL of the 5 x RO-Easy™ Mix and 6 μL of RNase-Free ddH_2_0 using Superscript first strand synthesis system (Abm, Canada). Stored the resultant cDNA at −20 °C.

### RNA-Seq library construction, sequencing, and analyses

RNA from six gill samples (3 samples per group) that met the sequencing requirements were selected and subjected to the RNA-Seq library construction using the TruSeq™ RNA Sample Prep Kit (Illumina, San Diego, CA, USA). The requirements were as follows: RNA quantity ≥ 1ug, RNA concentration ≥ 35ng/μL, OD260/280 ≥ 1.8, OD260/230 ≥ 1.0. Magnetic beads with Oligo (dT) were used for A-T base pairing with ployA to enrich mRNA. Randomly fractured the mRNA by fragmentation buffer, sifted out small fragments of about 300bp. Subsequently, under reverse transcriptase, random hexamers were added to synthesize first-strand cDNA using mRNA as template, followed by second-strand synthesis to form a stable double strand structure. These double-stranded cDNA fragments were end-repaired by adding a single “A” base and ligation of adapters. The six libraries were sequenced using an Illumina Novaseq 6000 platform, with 150bp pair-end reads produced.

The clean reads were obtained by trimming adaptor sequences, filtering out reads with N and short reads with lengths of less than 10bp, as well as low-quality reads. Then the high-quality mRNA reads were mapped back to the goldfish (*Carassius auratus Linnaeus*) genome (https://www.ncbi.nlm.nih.gov/genome/?term=txid7957[orgn]), using TopHat2 (http://tophat.cbcb.umd.edu/). The software Cufflinks (http://cole-trapnelllab.github.io/cufflinks/) was used to assemble the mapped reads. The unigenes were compared with known transcripts from six databases to obtain annotated information, including Gene Ontology (GO), Kyoto of Encyclopedia of Genes and Genomes (KEGG), Clusters of Orthologous Groups of proteins (COG), National Center for Biotechnology Information (NCBI) non-redundant protein sequences (NR), Swiss-Prot, and The Pfam protein families (Pfam). The expression level of the transcript was quantitatively analyzed by RSEM software (http://deweylab.github.io/RSEM/). FPKM (Fragments Per Kilobases per Millionreads) was used to analyze the expression levels of differential genes. DESeq2 algorithms was used to select a subset of differentially expressed genes (DEGs), and p-values were adjusted using Benjamini-Hochberg’s approach. Finally, genes with adjusted P ≤ 0.05 and |log2 (fold-change) | ≥1 were seen as DEGs and considered as the targets for further analyses.

### Histology, TUNEL, and immunohistochemical studies

#### Hematoxylin-eosin staining

Twelve gill samples (6 samples per group) were obtained for histopathological analysis. Briefly, the second and third gills on the right were removed with sterile forceps, immediately fixed in 4% neutral buffered formalin over 24h, dehydrated in a graded ethanol series (30%, 50%, 70%, 80%, 90%, and 100% × 2; 10 min each), cleaned in xylene, embedded in paraffin, subsequently sectioned at approximately 5 μm thickness (LeicaRM2125RTS, Lycra, Germany), and stained with classical hematoxylin and eosin (H & E). Tissue slices were examined using optical microscope (Nikon Eclipse E200, Japan). Histological changes in gill tissue including cell hyperaemia, lamellae fusion, cell hyperplasia, and necrotic and detachment of epithelial cells were assessed using a score (15) ranging from 1 to 7, depending on the extent of lesions: (1) unchanged; (3) mild; (5) moderate; and (7) severe. Total histopathological score was the sum of the above pathological scores.

#### TdT-mediated dUTP nick-end labeling

The second gill on the right side of twelve fish (6 samples per group) was fixed in 10% formaldehyde solution, then dehydrated, embedded in paraffin, and sectioned. Tissue slices were dewaxed and treated with a graded ethanol series. After PBS cleaning, permeated with protease K and then cleaned with PBS again. Subsequently, the labeling reaction was performed using a labeled solution containing terminal deoxynucleotide transferase, buffer, and fluorescein dUTP at 37 °C. Following incubation, excess labelling solution was washed off with PBS, then the slices were counterstained with hematoxylin, washed with tap water, dehydrated with gradient alcohol, transparent with xylene and sealed with neutral gum. Finally, the slices were observed with an inverted fluorescence microscope (Leica DMIRB, Germany) and captured with a CCD camera (Olympus CKX41, Japan), and the number of TUNEL positive cells (2mm^2^) was recorded. And the gill mean fluorescence intensity (2mm^2^) was assessed using ImageJ.

#### Immunohistochemical studies

The paraffin sections were dewaxed to water and then placed in a repair box filled with citric acid antigen repair buffer (pH 6.0) for antigen repair in microwave oven. Then, the slices were placed in 3% hydrogen peroxide solution, incubated at room temperature away from light for 25 min, and washed in PBS (pH 7.4) for 3 times, 5min each to block endogenous peroxidase. Then 3% BSA (Thermo Fisher, USA) was added to cover the tissue evenly and sealed at room temperature for 30min. The primary antibody Zap-70 (99f2-cell Signaling) Rabbit mAb (CST, Massachusetts, USA) equipped with PBS was added and incubated overnight in a wet box at 4°C. After washing, cover tissue with Goat Anti-Rabbit IgG (H&L) Alexa Flour 488secondary antibody (Thermo Fisher, USA), and incubate at room temperature for 50min. After DAB color rendering, restaining the nucleus, dehydrating and sealing, the sections were observed under Nikon Eclipse E200 (Japan), and photographed for analysis. Mean optical density (IOD SUM/area) was evaluated by image J and immunohistochemical scores (IHC score) were analyzed by IHC Profiler. The scoring standards are as follows: (3) strong positive; (2) positive; (1) low positive; (0) negative.

### Quantitative real-time PCR analysis

To analyze the expression of immune-related genes and apoptosis-related genes, as well as to validate sequence quantification, quantitative real-time PCR (qRT-PCR) was performed targeting some selected DEGs associated with immunity (*IL-1β, IL-10, IL-8*) and apoptosis (*Caspase 3, Bad, Bax*). As an internal control, the *β-actin* gene of goldfish was used to normalize the expression level of genes. qRT-PCR was performed in a total volume of 10 μL containing 5 μL of TB Green™ Premix Ex Taq™ II, 0.2 μL of Rox, 1 μL of cDNA, 0.8 μL of each primer (specific primers outlined in **Table 1**) and 2 μL of double distilled water. The reaction conditions used were as follows: 95°C for 3 min, followed by 39 cycles of 95°C for 10 s, 60°C for 20 s and 72°C for 20 s, with the dissolution curve increasing from 0.5°C to 95°C every 5 s. Ct values from goldfish gene expression were normalized to Ct levels of *β-actin*, and the relative expression of genes was estimated by the 2^–ΔΔCT^ method. The assays were performed on a real-time fluorescence quantitative PCR System (Bio-Rad, USA).

**Table 1:** Primers used for gene expression analysis by qRT-PCR in gill of goldfish.

Gene	Name	Forward Sequence	Reverse Sequence	Efficiency
*IL-8* XM_026204952.1	Interleukin 8	TGAGTCTTAGCGGTCTGGGTGT	GAGGGAAGAGCTCCACACTCTCT	95%
*IL-10* LC008369.1	Interleukin 10	CAAGGAGCTCCGTTCTGCAT	TCGAGTAATGGTGCCAAGTCATCA	95%
*IL-1β* AB757758.1	Interleukin 1β	GATGCGCTGCTCAGCTTCT	AGTGGGTGCTACATTAACCATACG	96%
*Bax* XM_026262399.1	Bcl-2-associated x protein	GCAGCAGATCGGAGATGAGC	GACAAGGCGACAGGCAAAGTAG	95%
*Bad* XM_026267190.1	Bcl2-associated agonist of cell death-like	CCTCTCACTTTGCCGCATTG	TCCCCTGGTCTTTTGGGTGT	96%
*Caspase 3* *XM_026203855.1*	caspase-3-like	AGGCTGACAGCTCCGATAGT	CAGGCTGGTTGGAGGTTGAC	94%
*β-actin* LC382464.1	β-actin	GATGCGGAAACTGGAAAGGG	ATGAGGGCAGAGTGGTAGACG	97%

### Statistical analyses

In this study, all data are presented as mean ± SD (standard deviation), Ct values from goldfish gene expression were normalized to Ct levels of *β-actin*, and the relative expression of genes was estimated by the 2^–ΔΔCT^ method. Statistical difference was analyzed by SPSS 27.0 software (IBM Corp., Chicago, USA). Charts were drawn by GraphPad Prism (USA) and Adobe Illustrator (USA) software. Significant difference was determined using the one-way ANOVA analysis, and the significant level was set as P < 0.05 (*), P < 0.01 (**) or P < 0.001 (***).

## Results

### Identification of *Myxobolus ampullicapsulatus*

**Figure 1:**
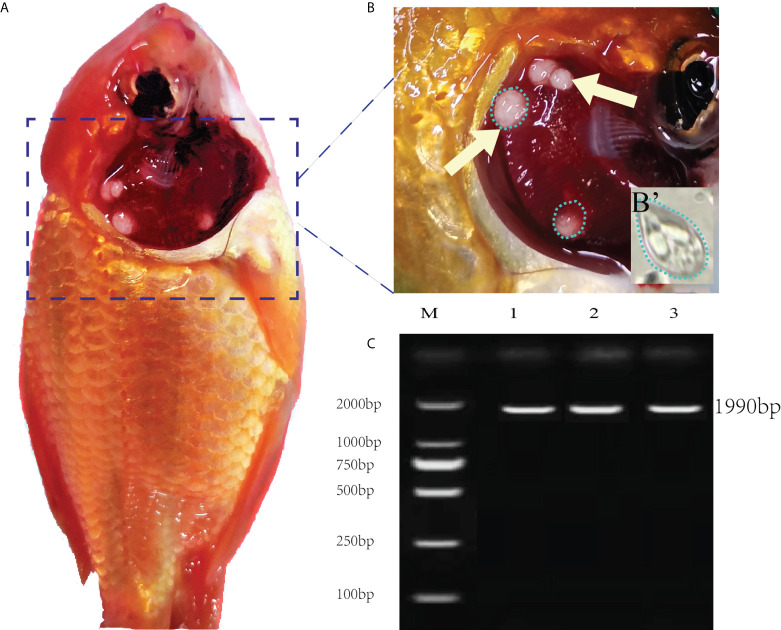
Clinical signs of diseased fish and electrophoretic results. **(A)** Clinical signs of goldfish infected with *Myxobolus ampullicapsulatus*. **(B)** Opalescent rounded cysts (dotted circle) of varying sizes appeared on the gills (arrows). (B’) Spore morphology under high amplification field with oil immersion lens, x100. **(C)** Electropherogram of *Myxobolus ampullicapsulatus*, M: marker. Lane 1-3: DNA of goldfish gill infected by *Myxobolus ampullicapsulatus*.

**Figure 2:**
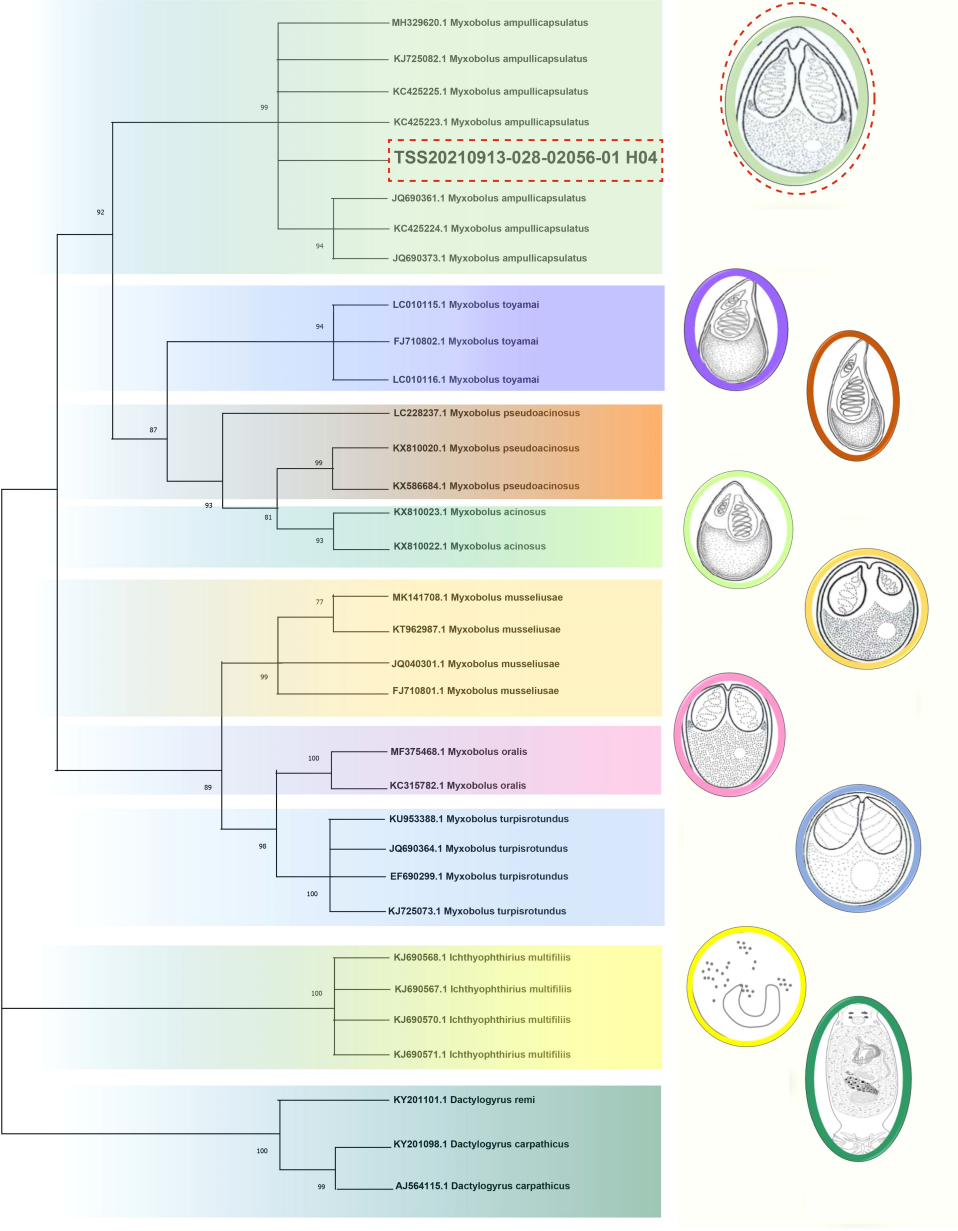
Phylogenetic trees based on 18S rDNA sequences using the NJ method with 1000 bootstraps. On the right are different types of *Myxobolus* and two foreign species (the *Ichthyophthirius multifiliis* and the *Dactylogyrus*). The same color represents the same species. The red dotted line represents the sequence and *Myxobolus ampullicapsulatus* pattern diagram of this study. The parasite pattern diagrams were refer from LiuYang (16), Eva Řehulková (17) and Luqi Tan (18).

The necropsy showed the diseased goldfish gill mucus increased with opalescent rounded cysts of varying sizes obviously distributing on the swollen gill filaments **(** **Figures 1A, B** **)**. The cyst contained a large number of spores. Each spore had a pear-shaped shell surface, containing two distinct bottle-shaped polar capsules. The polar capsules at the front of the spore were drawn close to one another, accounting for about two-thirds of the spore length **(** **Figure 1B** **)**. Electrophoresis results showed that stripe size obtained by amplification with specific primers of *Myxobolus* (5′- TTC TGC CCT ATC AAC TTG TTG-3′ and 5′- CTA CGG AAA CCT TGT TAC G-3′) was about 1900bp **(** **Figure 1C** **)**, which was 99% homologous with the sequence of *Myxobolus ampullicapsulatus* (KC425223.1) in GenBank. This sequence was phylogenetic analyzed together with 18S rDNA sequences of different *Myxobolus* obtained from the GenBank database, and was clustered into a clade with *Myxobolus ampullicapsulatus* **(** **Figure 2** **)**. Therefore, we identified this *myxobolus* as *Myxobolus ampullicapsulatus*.

**Figure 3:**
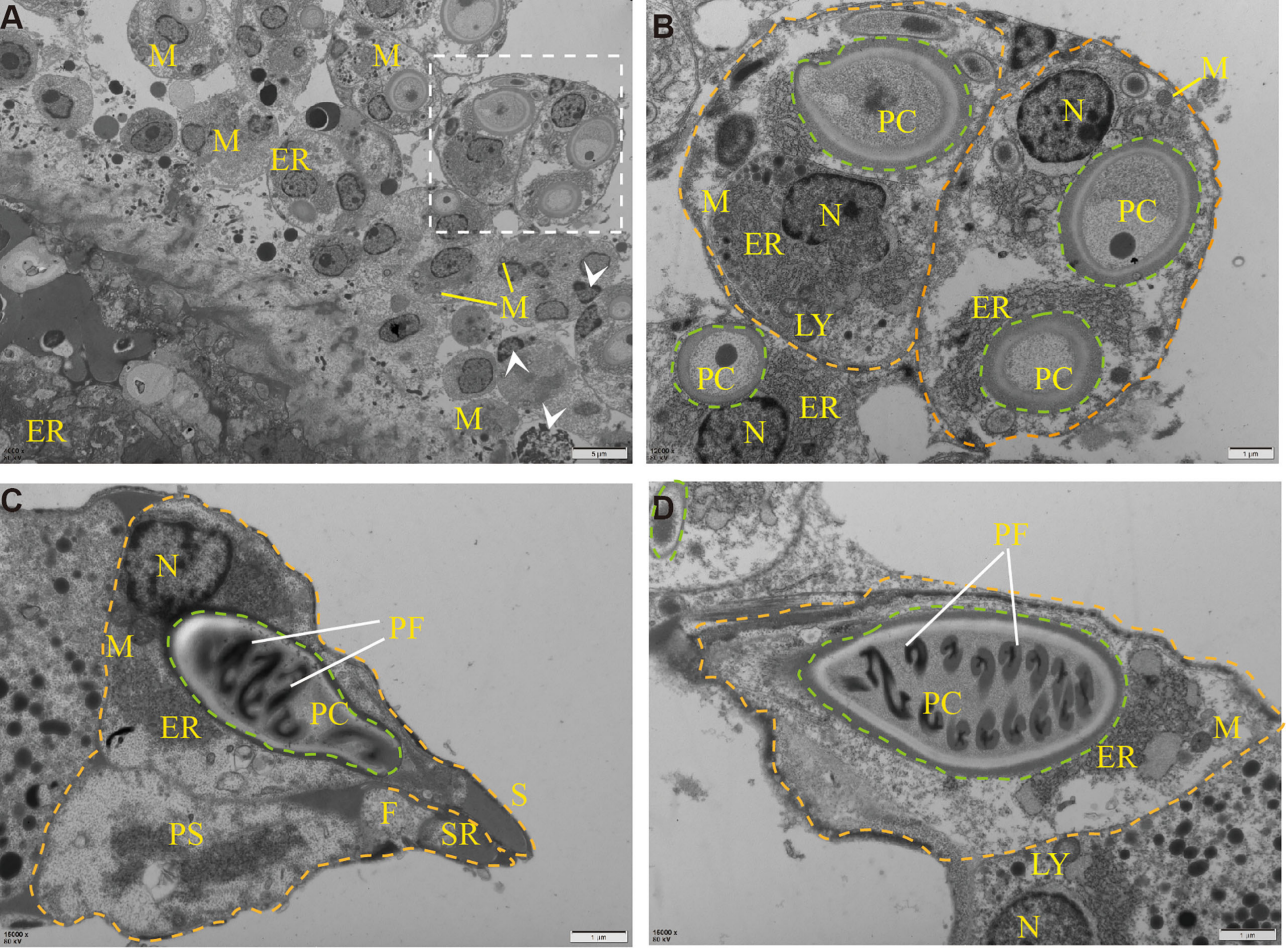
Transmission electron microscope observation of *Myxobolus ampullicapsulatus*. **(A)** Ultrapathological changes of goldfish infected with *Myxobolus ampullicapsulatus*, including mitochondrial and endoplasmic reticulum swelling, apoptosis (arrowhead), etc. **(B)** Partial enlargement of white dotted line. Transverse section of mature spore, each spore (orange dotted line) contained polar capsules at different stages (green dotted line) and was rich in organelles. **(C, D)** longitudinal section of the polar capsule: **(C)** The spore contained sporoplasm, a bottle-shaped polar capsule and was surrounded by a spore shell. **(D)** About 8 pairs of double “S” shaped polar filaments were wound in the polar capsule. PC, polar capsule. PS, sporoplasm. PF, polar filament. N, nucleus. M, mitochondria. LY, lysosome. ER, endoplasmic reticulum. F, faveolate electron-dense object. SR, sutural ridges. S, scabbard. arrowhead, apoptotic cell. Orange dotted line: spore. Green dotted line: polar capsules.

To determine the ultrastructure of *Myxobolus ampullicapsulatus*, electron microscope observation was carried out, and the results showed that the spore mainly consists of sporoplasm, spore shell, and polar capsule, which was consistent with the results of other studies (19). A large number of mitochondria, rough endoplasmic reticulum, lysosome and other organelles as well as some vesicular structures and proenzyme granules can be observed in sporoplasm **(** **Figure 3B** **)**. The spore shell was smooth and composed of two shell valves which were symmetrically thickened at the sutural ridges. Besides, there were scabbard-like structures in the depression of the shell valves toward sutural ridges and faveolate electron-dense object at the base of it **(** **Figure 3C** **)**. The polar capsule was bottle-like and located at the front of the spore. Winding externally along the polar capsule, was the double “S” shaped polar filaments **(** **Figure 3D** **)**. These indicate that the parasite is equipped with abundant and impeccable organelles. In addition, mitochondria and endoplasmic reticulum swelling, and cell apoptosis **(** **Figure 3A** **)** were also observed, indicating *Myxobolus ampullicapsulatus* caused pathological changes in gill tissue of goldfish.

### Histological effects of *Myxobolus ampullicapsulatus* on gills of goldfish

**Figure 4:**
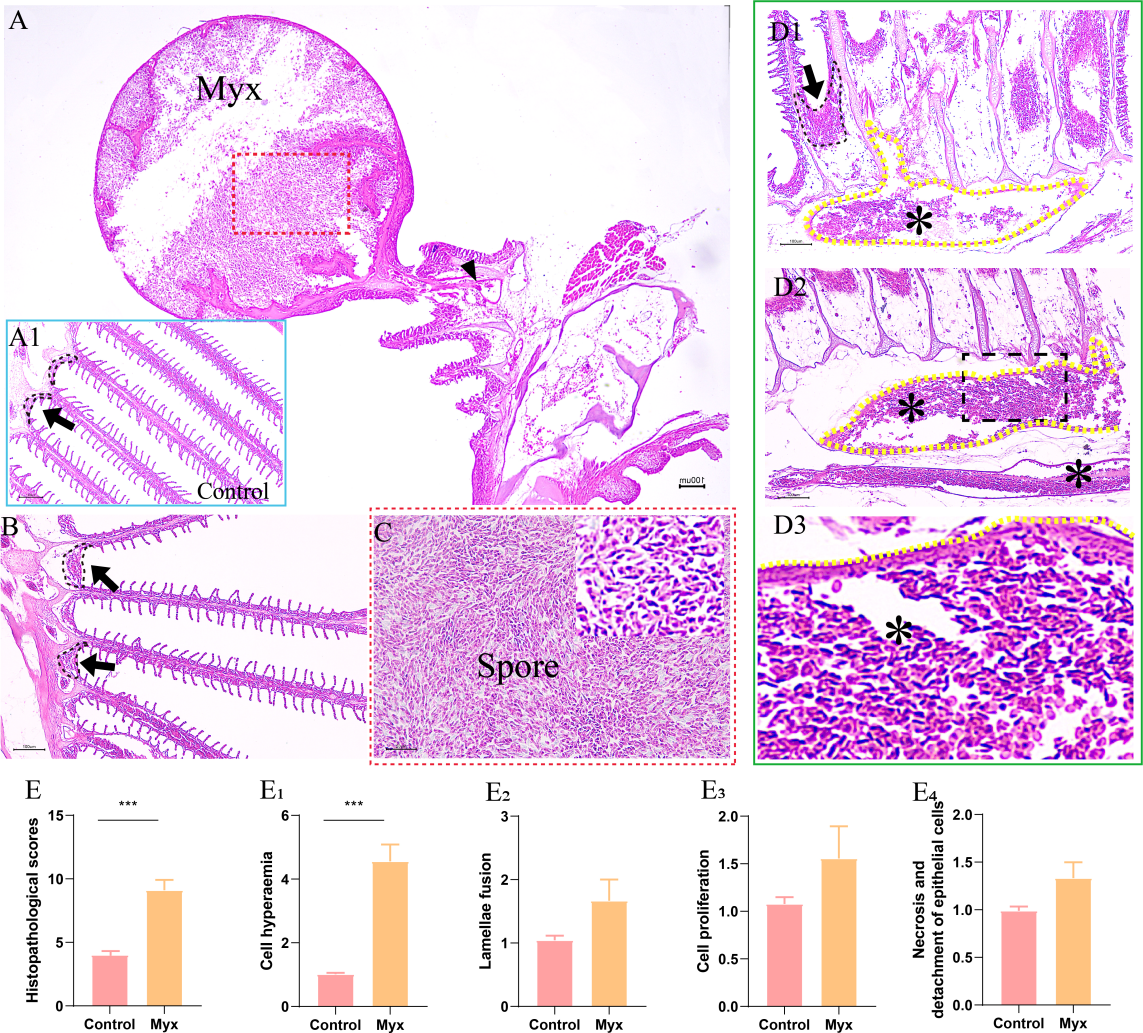
Histopathological observation of gills of goldfish infected with *Myxobolus ampullicapsulatus*. **(A)** Round cysts attaching to gill filaments contained a large number of spores (red dotted box), some of which released from the cysts and entered the gill tissue through the branchial artery (black triangle). (A1) Control group goldfish, with neatly arranged gill lamellae, showing continuous gill-associated lymphoid tissue (black dotted line and black arrow) formed on the gill arch. **(B)** The gill-associated lymphoid tissue in Myx group was dilated (black dotted line and black arrow). **(C)** Partial enlargement (red dotted box) of Figure A, dense spores can be seen. **(D)** The gill of Myx goldfish showed obvious hyperemia (*) (P value), accompanied by a large number of spores distributed in the afferent branchial artery (yellow dotted line) **(E)** Total score of gill histopathological score; (E_1_–E_4_) The histopathological scores for cell hyperaemia, lamellae fusion, cell hyperplasia, and necrotic and detachment of epithelial cells, respectively. Significance: P < 0.05 (*), P < 0.01 (**), P < 0.001 (***). (n=6).

By observing the pathological section of gill tissue at the parasitic site of the cyst, it can be seen that the cyst was mainly parasitic in the interlamellar region of goldfish gill filament **(** **Figure 4A** **)**. The cyst expanded into a round shape and contained a large number of mature spores **(** **Figure 4C** **)**, some of which flew out of the cyst to form a cavity and entered the gill tissue through the branchial artery **(** **Figure 4A** **)**. Compared with the control group **(** **Figure 4A1** **)**, the Myx group showed that the gill-associated lymphoid tissue on both sides of the gill arch was dilated **(** **Figure 4B** **)**, the gills were obviously hyperemic, accompanied by a large number of spores distributed in the afferent branchial artery **(** **Figure 4D** **)**, indicating that the parasite can infect goldfish through the circulatory system. However, the morphology of gill lamella in Myx group was comparatively normal with relatively orderly arrangement. No obvious adaptation and repair process such as gill lamella hyperplasia and cell fusion were observed **(** **Figure 4B** **)**. In brief, no significant deformation or inflammatory response was observed in gill tissue after parasite infection, except for severe gill filament hyperemia **(** **Figure 4E** **)**.

### Analysis of transcriptomic changes in goldfish gill after *Myxobolus ampullicapsulatus* infection

Illumina Novaseq 6000 sequencing platform was used to complete transcriptomic sequencing of six samples (three samples in each group), and a total of 61.95 Gb of Clean Data were obtained. Clean Data of each sample was over 6.27 Gb, and the percentage of Q30 base was over 93.93% **(** **Table S1** **)**. Clean Reads of each sample were sequentially compared with the specified reference genome, with alignment rates ranging from 80.29% to 91.58%. Based on analysis, a total of 64783 expressed genes were detected, including 59328 known genes and 5455 new genes. A total of 127,254 expressed transcripts were obtained, including 95289 known transcripts and 31965 new transcripts. By using variance analysis software DESeq2 (screening threshold for: |log2FC| ≥1, padjust<0.05), differentially expressed genes were analyzed based on quantitative results of expression levels, and a total of 1169 DEGs were obtained between the two groups, including 577 up-regulated genes and 592 down-regulated genes. All genes and transcripts obtained by transcriptomic assembly were compared with the six databases (NR, Swiss-Prot, Pfam, EggNOG, GO and KEGG), and the annotations in each database were shown in **Table 2**.

**Table 2:** Annotation summary of goldfish gill tissue transcriptome.

Database-Annotated	Number of annotated unigenes	Percentage of annotated unigenes (%)
Annotated in Go	25645	43.23
Annotated in KEGG	37956	63.98
Annotated in COG	54909	92.55
Annotated in NR	56985	96.05
Annotated in Swiss-Prot	50573	85.25
Annotated in Pfam	47118	79.42
Total annotated	57054	96.17

**Figure 5:**
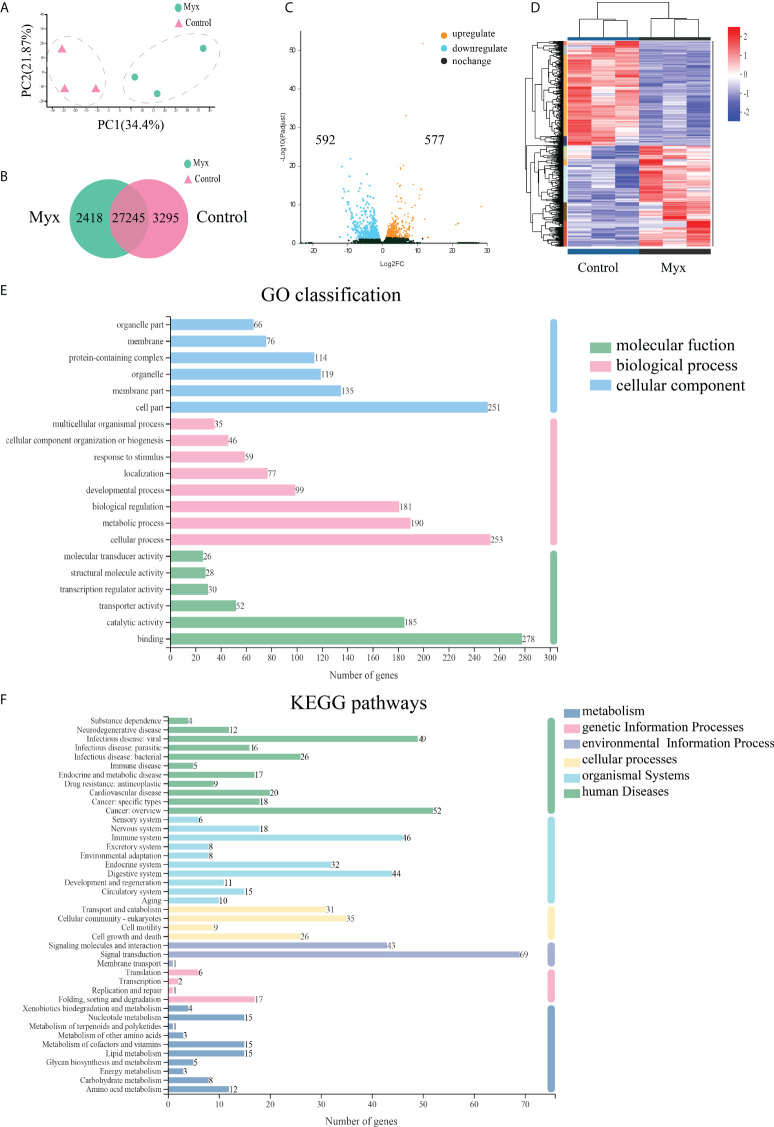
Effects of *Myxobolus ampullicapsulatus*-stimulation on the transcriptomic dynamic changes of goldfish gill and DEGs classification. **(A)** Principal component analysis (PCA) of the gill transcriptome in basal conditions and Myx-infected groups. **(B)** Venn diagram of DEGs among control and Myx groups. **(C)** Volcano plot displaying the DEGs distribution in two groups. **(D)** Hierarchical clustering analysis divided the individual samples of two groups based on FPKM of DEGs. Red and blue indicated that the gene expression level was up-regulation and down-regulation, respectively. **(E)** Histogram of GO annotation analysis. Bule bars, cellular component; pink bars, biological process; green bars, molecular function. **(F)** Histogram of KEGG annotation analysis. Green bars, human diseases; light bule bars, organismal systems; yellow bars, cellular processes; lavender bars, environmental information processing; pink bars, genetic information processing; deep blue bars, metabolism.

Prior to DEGs analysis, principal component and inter-sample venn analysis were performed based on the expression matrix. PCA plot showed significant differences between the control and Myx groups **(** **Figure 5A** **)**. From the venn plots, there were 2418 unique genes in the Myx group, 3295 unique genes in the control group, and 27245 common genes expressed in both groups **(** **Figure 5B** **)**. Volcano plot **(** **Figure 5C** **)** and hierarchical clustering analysis **(** **Figure 5D** **)** displayed the DEGs distribution between the two groups, indicating that there were significant differences in gene expression. The DEGs were then annotated by GO, and the results showed that 2452 unigenes were classified into 45 GO terms, of which “Cell part”, “Cellular process”, and “Binding” were dominant in the categories “Cellular component”, “Biological process”, and “Molecular function”, respectively **(** **Figure 5E** **)**. To investigate the potential pathways involved in physiological function of gill tissues infected with *Myxobolus ampullicapsulatus*, 747 DEGs were classified into 42 KEGG pathways, of which the pathway with most annotated unigenes was “Signal transduction” (69 unigenes), followed by “Cancers overview” (52 unigenes), “Infectious diseases-viral” (49 unigenes) and “Immune system” (46 unigenes) **(** **Figure 5F** **)**.

**Figure 6:**
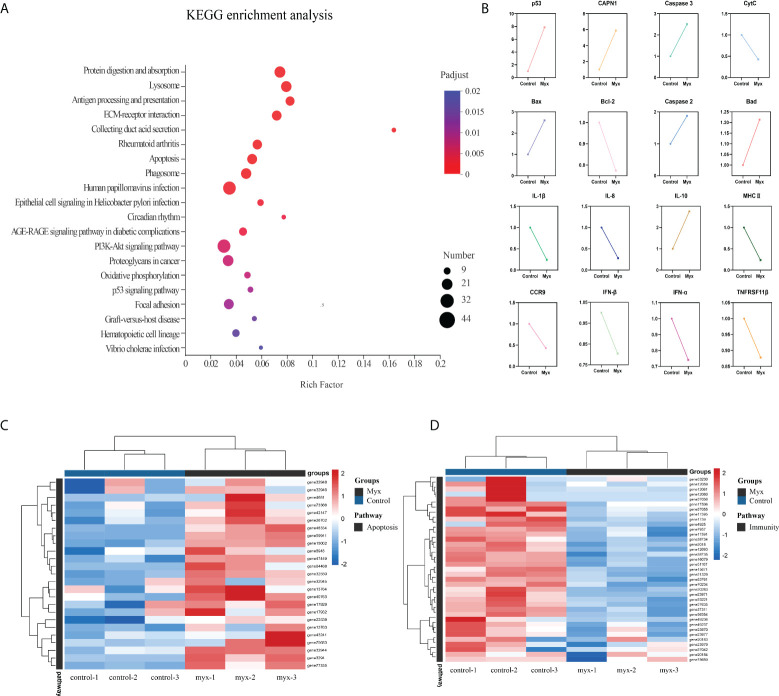
Enrichment analysis and cluster analysis of goldfish gill tissue transcriptome. **(A)** Scatter plots of enriched gene ontology biological process (BP) terms for gill tissue in two groups using the KEGG database. The fold enrichment indicates the ratio of the expressed gene number to the total gene number in a pathway. Only the top 20 BPs according to p-value were shown. The size and color of the points represent the gene number and the log10 p-value of each pathway, respectively. **(B)** Expression patterns of DEGs associated with apoptosis and immunity in the KEGG enriched pathway. **(C)** Heatmap of apoptosis-related DEGs. **(D)** Heatmap of immune-related DEGs. Red and blue indicated the up regulated and down-regulated gene, respectively. The scale of the color bar represented fold changes of gene expression. Myx 1–3 represent gill tissue samples with *Myxobolus ampullicapsulatus* infection. Control 1–3 represent gill tissue from healthy fish without parasite infection.

To further investigate the DEGs which were involved in responding to *Myxobolus ampullicapsulatus* infection between two groups, KEGG pathway enrichment analysis was conducted by scripting with R language, and the pathway was considered to be significantly enriched when P < 0.05. Analysis of the top 20 significant enrichment pathways showed that immune-related aspects such as “Lysozyme”, “Antigen processing and presentation”, “ECM-receptor interaction”, “Phagosome”, as well as apoptosis related aspects such as “p53 signaling pathway” and “Apoptosis” were the main enrichment pathways **(** **Figure 6A** **)**. The results showed that the immune response of gill tissue of Myx goldfish was different from that of control, and the apoptosis pathway was activated. Besides, through the intersection of the KEGG enrichment pathway, we identified differential expression of immune-related genes, as well as apoptosis-related genes dominated by the p53-Bcl2/Bax signaling pathway in *Myxobolus ampullicapsulatus* infected goldfish **(** **Figure 6B** **)**. Cluster analysis of DEGs associated with immunology and p53-Bcl2/Bax mediated apoptosis showed that there were significantly different expression patterns between the control group and the Myx group. Compared with the control group, most apoptosis-related genes **(** **Figure 6C** **)** were significantly up-regulated in the Myx group, while most immune-related genes **(** **Figure 6D** **)** were observably down-regulated. Therefore, the death of goldfish induced by *Myxobolus ampullicapsulatus* may be related to immunosuppression and p53-Bcl2/Bax mediated apoptosis pathways.

### RNA-Seq validation using qRT-PCR

**Figure 7:**
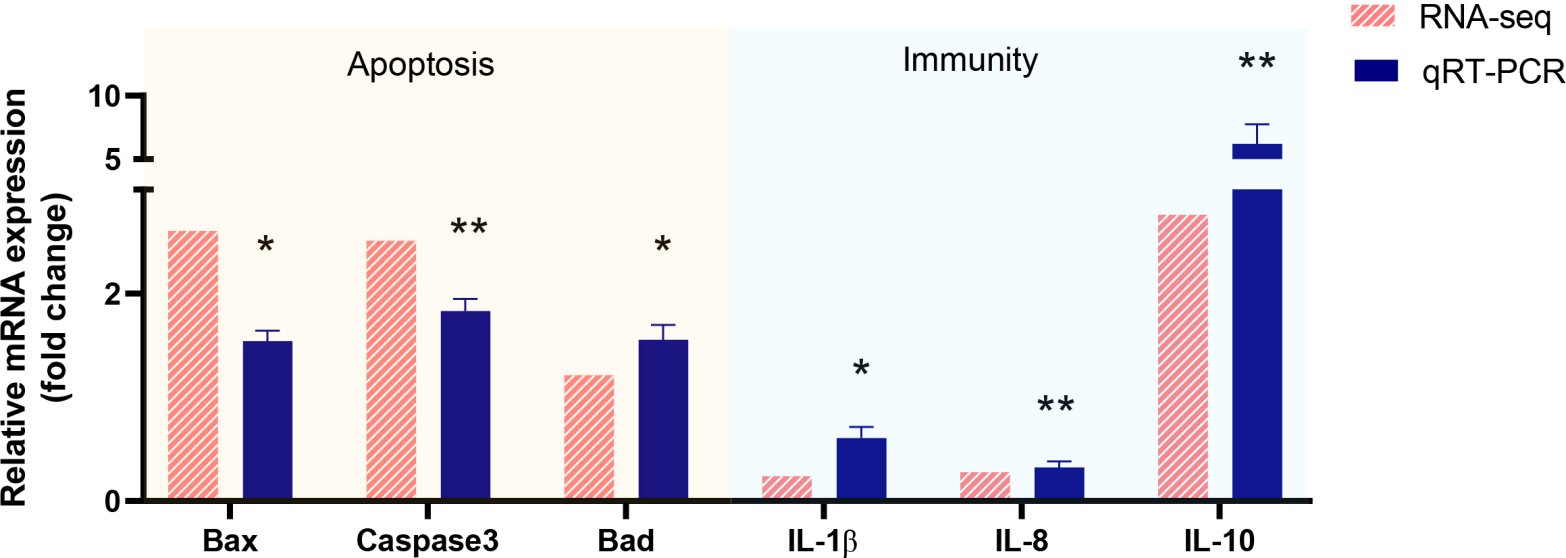
Validation of RNA-seq data by using real-time qRT-PCR. P < 0.05 (*), P < 0.01 (**), P < 0.001 (***).

To verify the validity and accuracy of transcriptome sequencing and explore the effects of *Myxobolus ampullicapsulatus* infection on the immunity and apoptosis of goldfish gill, we selected 6 DEGs (3 immune-related genes and 3 p53-Bcl2/Bax mediated apoptosis-related genes) for qRT-PCR. As shown **Figure 7**, The relative gene expression fold change trend obtained by transcriptome sequencing and qRT-PCR was consistent, indicating the accuracy and reliability of transcriptome sequencing data. The expression levels of key genes mediating apoptosis (*Bad*, *Bax*, Caspase*3*) were significantly higher in the Myx group than control group, indicating that apoptosis was strongly induced in the gills of parasites infected goldfish. In addition, cytokines promoting inflammation such as *IL-8* and *IL-1β*, were significantly down-regulated in the Myx group, while anti-inflammatory cytokine *IL-10* was markedly up-regulated, suggesting that inflammation was suppressed in the gill tissue infected with parasites, which may lead to immunosuppression.

### TUNEL and immunohistochemical analyze in goldfish gill after *Myxobolus ampullicapsulatus* Infection

**Figure 8:**
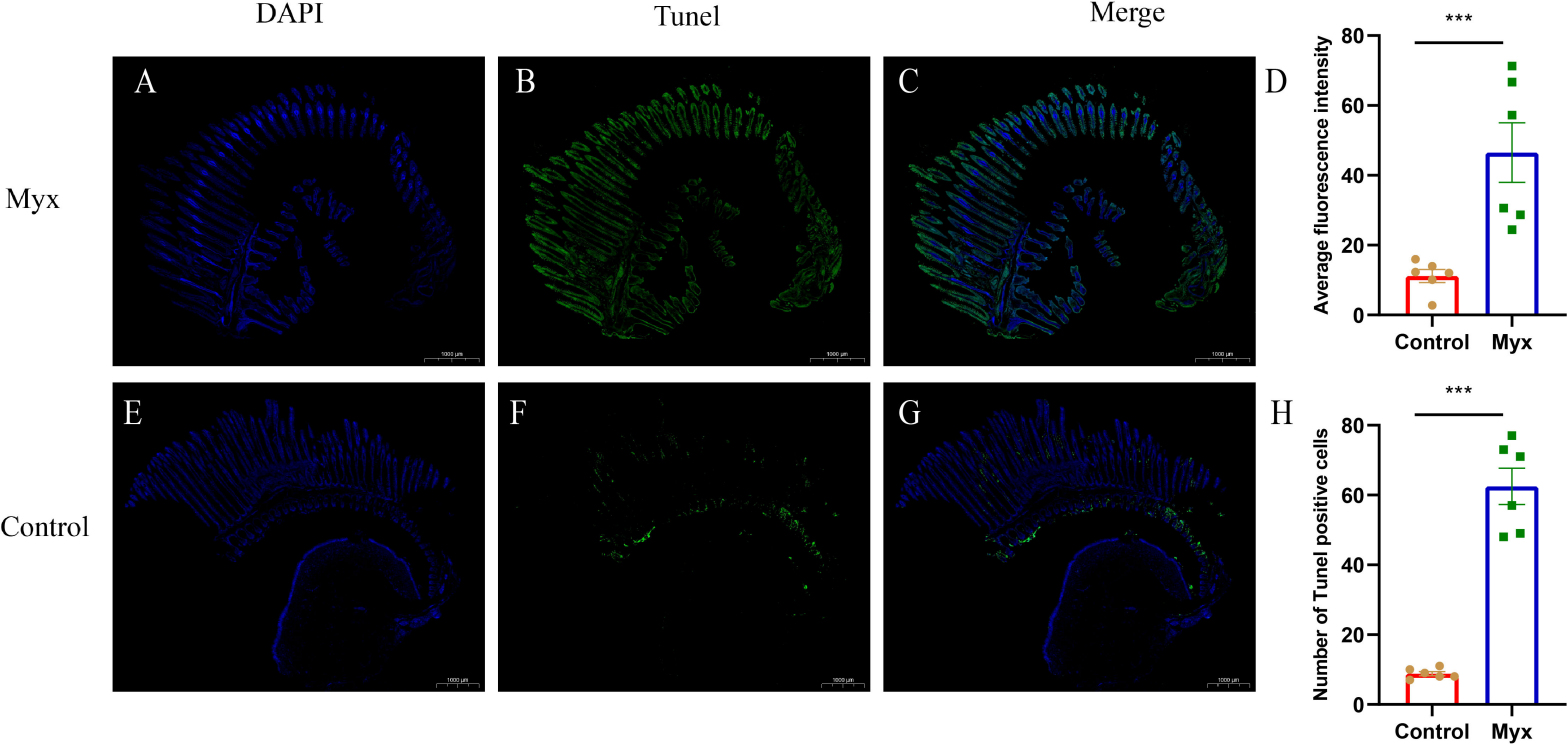
TUNEL assays of gills which were stained with DAPI (blue) or fluorescein-dUTP (green). **(A–C)** TUNEL assays of goldfish in Myx group, Plentiful apoptotic cells appeared on the gill of Myx group. **(E–G)** TUNEL assays of goldfish in control group, very few apoptotic cells presented on the gill of control group. **(D)** Statistical analysis of mean fluorescence intensity (2mm^2^). **(H)** Statistical analysis of TUNEL positive cells numbers (2mm^2^). Significance: P < 0.05 (*), P < 0.01 (**), P < 0.001 (***). (n=6).

To investigate whether *Myxobolus ampullicapsulatus* infection could cause apoptosis of gill tissue in goldfish, TUNEL assay was performed. The gill of the Myx group showed a large number of positive signals of apoptotic cells labeled by fluorescein-dUTP **(** **Figures 8A–C** **)**, indicating that the gill of the Myx group suffered severe apoptosis. In contrast, very few positive apoptotic signals were observed in the control group **(** **Figures 8 E–G** **)**. Statistical analysis showed that the Myx group had significantly higher mean fluorescence intensity **(** **Figure 8D** **)** and notably greater number of apoptotic cells **(** **Figure 8H** **)**.

**Figure 9:**
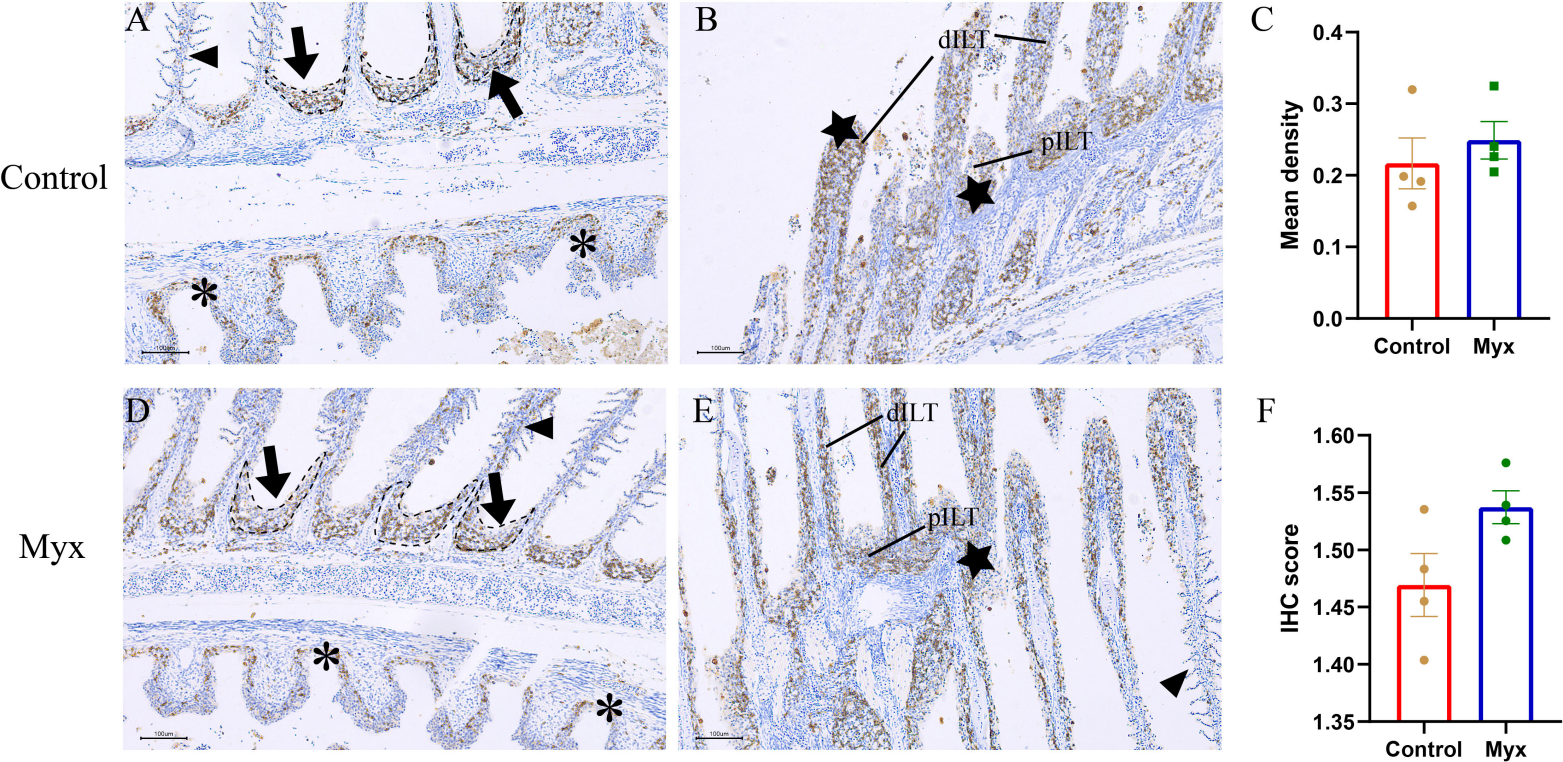
Representative immunofluorescent images of gills which were stained with Zap-70^+^ (brown). **(A, B)** Immunohistochemical analysis of goldfish in control group: **(A)** A few Zap-70^+^ cells appeared on ALT (black dotted line, black arrow), gill arch (*), and gill filament (black triangle). **(B)** Plentiful Zap-70^+^ cells appeared on distal ILT (dILT) and proximal ILT (pILT) (★). **(C)** Statistical analysis of mean optical density. **(D, E)** Immunohistochemical analysis of goldfish in Myx group: **(D)** A small number of ZAP-70^+^ cells were distributed in ALT (black dotted line, black arrow), gill arch (*), and gill filament (black triangle). **(E)** Plentiful Zap-70^+^ cells appeared on ILT (★). **(F)** Statistical analysis of IHC score. Significance: P < 0.05 (*), P < 0.01 (**), P < 0.001 (***). (n=4).

To investigate whether *Myxobolus ampullicapsulatus* infection could cause immunosuppression of gill tissue in goldfish, immunohistochemical analysis was conducted. T/NK cells, positive signals of ZAP-70, were observed in both the control group **(** **Figures 9A, B** **)** and Myx group **(** **Figures 9D, E** **)**, mainly distributed in ILT (Interbranchial lymphoid tissue) and ALT (Amphibranchial lymphoid tissue) (20), and partially dispersed in the gill arch and gill lamella. However, there was no significant difference in the mean optical density **(** **Figure 9C** **)** nor IHC score (Immunohistochemistry score) **(** **Figure 9F** **)** of gill tissue between the two groups, suggesting that infection with *Myxobolus ampullicapsulatus* could lead to immunosuppression of gill tissue to a certain extent without the increase in the number of gill lymphocytes.

### Potential regulatory mechanisms in *Myxobolus ampullicapsulatus* infected goldfish

**Figure 10:**
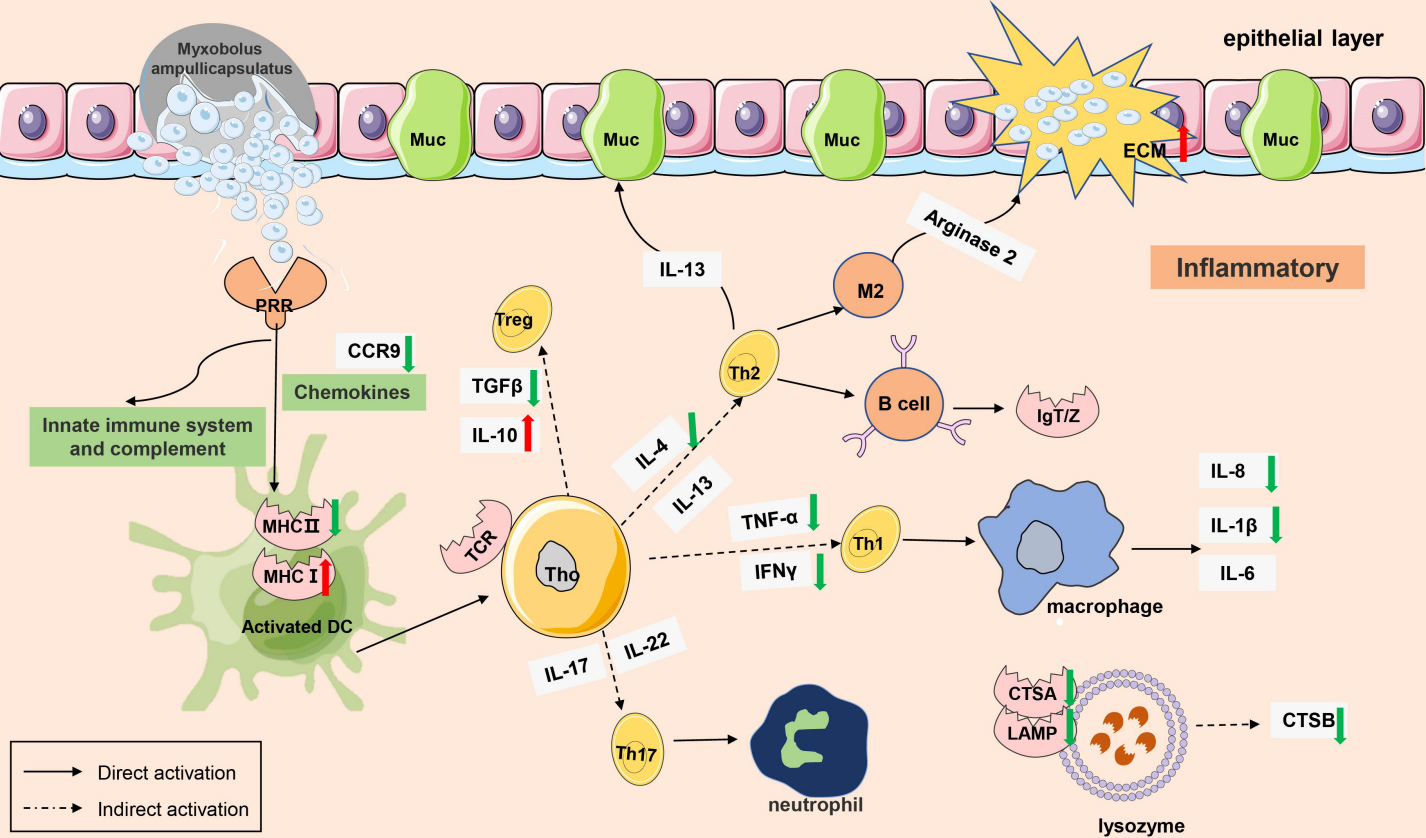
** **A model of immunosuppression in gills of goldfish infected with *Myxobolus ampullicapsulatus.* Schematic diagram showing the main immune-related events involved in gill *Myxobolus ampullicapsulatus* in goldfish inferred from the results of this study. Red arrow represents up-regulation; green arrow represents down-regulation.

**Figure 11:**
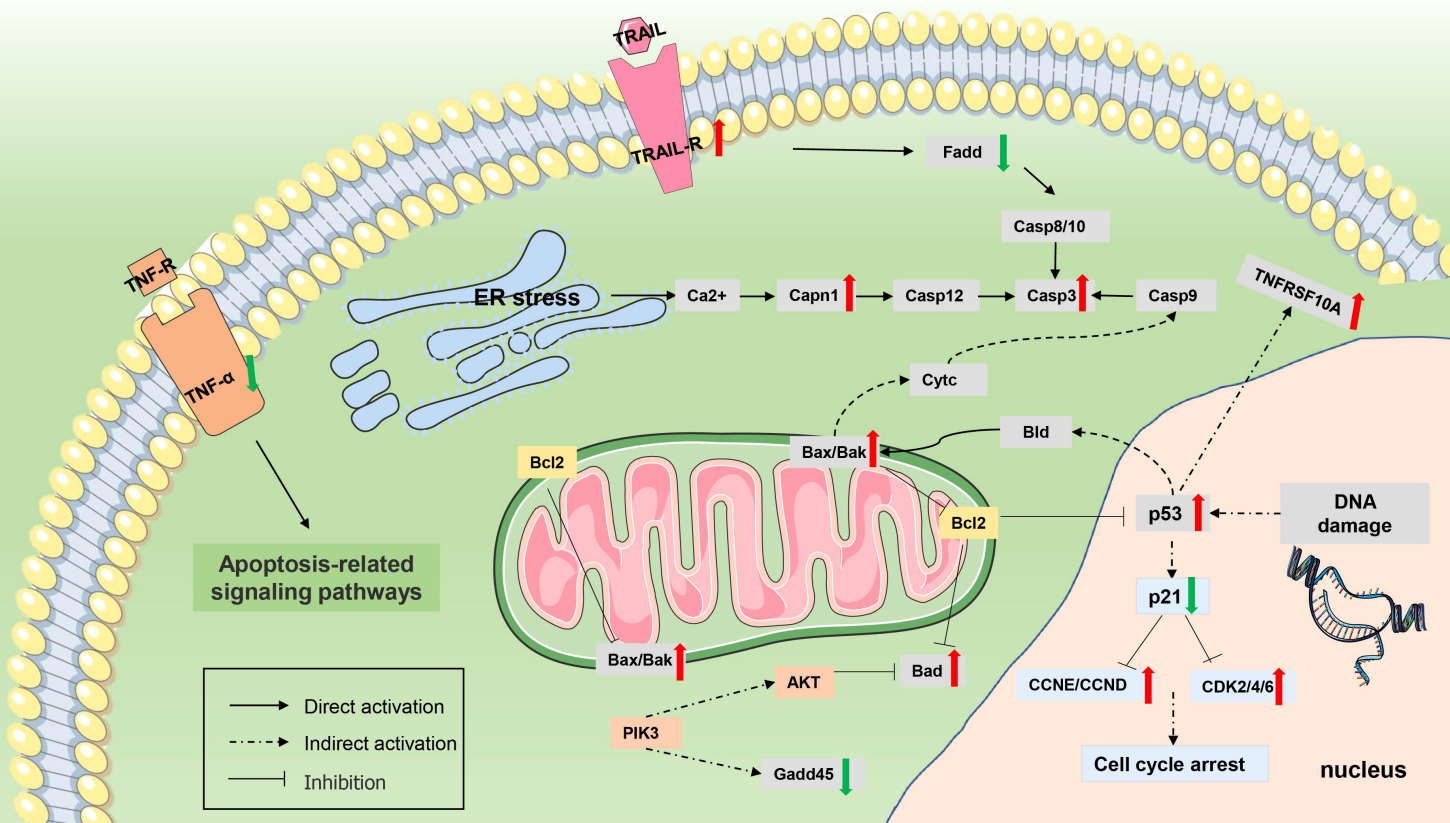
Differentially expressed genes (DEGs) involved in apoptosis. The genes whose expression was up-regulated (P value <0.05 and |log2(fold change)≥ 1) are highlighted in red, and whose expression was down-regulated (P value <0.05 and |log2(fold change)≥ 1) are highlighted in green.

The gene regulatory network of *Myxobolus ampullicapsulatus* infected goldfish was mapped according to KEGG enrichment pathways (**Figures 10**, **11** **)**. After the parasites adsorbed on the gill mucosal surface, a large number of mature spores were released to infect the gill tissue and induced the interaction between pattern recognition receptors (PRRs) and pathogen-associated molecular patterns (PAMP), leading to the activation of innate immune system, complement system and chemokines, which further transmitted intercellular signals to Th cell and B cell *via* antigen-presenting cells. Arginase 2 might promote extracellular matrix (ECM) deposition to repair tissue damage or encapsulate parasites. However, immune-related genes such as *IL-8, IL-1β, TNF-α, IFN-γ* and cathepsin in lysozyme were significantly down-regulated, and anti-inflammatory factor *IL-10* was notably up-regulated. This may be due to the that *IL-10* antagonizes the induction of proinflammatory mediators by PAMPS, leading to in immunosuppression of gill tissue through variety of mechanism interactions.

In addition, *Myxobolus ampullicapsulatus* infection might leads to DNA damage in gill tissue, resulting in increased p53 expression, further promoted Bak oligomerization and enhanced mitochondrial outer membrane permeability, and strongly facilitated the release of cytochrome C from healthy, stress-free mitochondria, causing cell apoptosis through the mitochondrial pathway. The release of cytochrome C also indirectly induced the increase of initial Caspase 9 expression, which then promoted the activation of executioner Caspase 3 signaling pathway, leading to cell apoptosis.

## Discussion

Histological and phylogenetic analysis revealed that the pathogeny of myxoboliosis in this study was *Myxobolus ampullicapsulatus* and enriched its morphological characteristics. Subsequently, transcriptome studies were used to reveal the regulatory mechanisms of goldfish gill disease caused by *Myxobolus ampullicapsulatus*, and the results showed that Myx goldfish gills were characterized by apoptosis activation dominated by p53-Bcl2/Bax signaling pathway and down-regulated immunity. Subsequent TUNEL assay, immunohistochemistry and qRT-PCR revealed severe gill apoptosis and suppressed gill immunity, further verifying the transcriptome results.

Apoptosis refers to the spontaneous and orderly cell death controlled by genes in order to maintain the stability of the internal environment (21). It is the process through which damaged or superfluous cells self-eliminate. Contrary to cell necrosis, apoptosis is an active process characterized by cytoplasmic concentration, nuclear membrane and nucleolus fragmentation, accompanied by the appearance of apoptotic bodies, but usually does not cause inflammation in surrounding tissues (22, 23). However, cell necrosis, a passive process, is defined as the disordered cell death caused by strong biological or physicochemical disturbances, often characterized by cell swelling, membranolysis, extravasation of cytoplasmic contents, accompanied by severe local inflammation (24, 25). In this study, under transmission electron microscope, cell shrinkage, agglutination and shift of nuclear chromatin, as well as swelling of mitochondria and endoplasmic reticulum were observed. These characteristics resemble typical features of gills apoptosis in puffer fish due to salinity fluctuations (26) and central nervous system apoptosis in sturgeon due to hypoxia (27), indicating there was severe apoptosis in gills of parasite infected goldfish.

Apoptosis can be divided into external pathways caused by the interaction of transmembrane receptors of the tumor necrosis factor family such as *TNFR/FAS* and associated ligands such as *TRAIL/Apo2* (28), and internal pathways involved in the *Bcl-2* family caused by mitochondrial damage (29). In the internal apoptotic pathway, the imbalance of pro-apoptotic proteins like BaX/BaK and anti-apoptotic protein Bcl-2 induces increased mitochondrial permeability, resulting in mitochondrial membrane potential disturbance and cytochrome C release (30). Both internal and external apoptosis channels, can lead to caspase-induced apoptosis execution pathways, which is primarily characterized by high activity of caspase enzyme, particularly caspase3 (31). According to transcriptome sequencing, “p53 signaling pathway” and “Apoptosis” were among the top 20 pathways highly enriched by KEGG in this study. Additionally, the swelling of mitochondria and the appearance of a significant number of lysosomes in the gill tissue of the goldfish infected with *myxobolus ampullicapsulatus* were observed under transmission electron microscopy. Besides, TUNEL analysis also revealed striking gill tissue apoptosis. In light of the aforementioned findings, it is possible that *myxobolus ampullicapsulatus* may lead to significant internal apoptosis of goldfish gill tissue.

P53 signaling pathway regulates various cellular reactions related to cell cycle (32), senescence (33, 34), apoptosis (35), DNA repair (36), and autophagy (37). As a core participant in regulating apoptosis, P53 protein can trigger caspase signaling pathway by inducing multiple apoptotic target genes (such as *Bcl-2*, *Bax*, *Bad* and *cytochrome C*), thus playing a direct or indirect role in multilevel apoptosis regulation (38, 39). Under normal circumstances, *p53* expression remains low. However, in response to DNA damage, hypoxia and viral infection, *p53* activity increased in damaged cells, resulting in cell cycle arrest or inducing Bak oligomization and a sharp increase in mitochondrial outer membrane permeability, as well as strongly promoting the release of cytochrome C, ultimately leading to apoptosis (40). Mice lacking *p53* showed reduced levels of apoptosis, defective antioxidant defense systems, and increased susceptibility to cancer (41). Deletion of *p53* leads to significant inhibition of human apoptosis pathway, resulting in schistosomiasis and schistosomiasis associated bladder cancer (BAC) (42). Similar to mammals, *p53* may also play an important role in the regulation of apoptosis in fish. Studies have shown that zebrafish (*Danio rerio*) promotes *Edwardsiella piscicida* infection through accumulation of *p53* in a transcription-dependent/-independent manner (43). Ketamine exposure induces expression of *p53* mediated apoptosis pathway in killifish (44). Giant seaperch iridovirus (GSIV) induces apoptosis through *p53*-mediated up-regulation of *Bax* and down-regulation of *Bcl-2* (45). In addition, the up-regulation of *Bax* and down-regulation of IFN-α/γ by p53 inhibition mediates the hyperproliferative response of Atlantic salmon (*Salmo salar L.*) gill epithelial cells with amoebic gill disease (AGD) (46, 47). For *Myxosporean*, it has previously been reported that sea bream (*Sparus aurata L*.) can induce a severe apoptosis response and cause severe desquamative enteritis after being challenged by *Enteromyxum leei* (48). Similar outcomes of fish tissue apoptosis caused by *myxosporean* were also discovered in this study. According to the up-regulation of *Bad* and *Bax* and the enhanced expression of *Caspase3* in gill of goldfish infected with *Myxobolus ampullicapsulatus*, the p53-Bcl2/Bax -related pathway was substantially activated and severe gill tissue apoptosis occurred.

Interleukins are subgroups of cytokines that regulate the fish immune system and are highly sensitive to pathogen infection and inflammation (49). Of particular concern are the pro-inflammatory cytokines *IL-1β* and *IL-8* released by immune cells, which are considered indicators for evaluating innate immune responses to pathogens and other stresses in fish (50). *IL-1β*, a typical multifunctional cytokine, is one of the most important mediators of host inflammation, affects practically all cell types (51). *IL-8* is a potent pro-inflammatory chemokine that regulates inflammatory response and phagocytosis by luring leukocytes to inflammatory and pathogen sites (52). *IL-10*, a regulator of inflammation and immunosuppression, activates JAK-STAT and PI3K-Akt signaling pathways and inhibits NF-κB signaling pathway by binding to specific receptor complexes. Numerous immunosuppressive effects of *IL-10* have been demonstrated, including reducing the expression of proinflammatory cytokines such as *IL-1*, *IL-8* and *TNF-α*, blocking the expression of major histocompatibility complex (MHC) class II and costimulatory molecules such as CD80/86, inhibiting TH1-mediated responses, and suppressing monocyte/macrophage function. (53–55). PAMPS can stimulate antigen-presenting cells such as monocytes/macrophages and dendritic cells to produce a variety of proinflammatory mediators that are essential for initiating immune responses, including cytokines and chemokines (56). However, *IL-10* can greatly antagonize the induction of proinflammatory mediators by PAMPS and inhibit the occurrence of immune responses, as *IL-10* can inhibit most of the key first-line defense cytokines *(TNF-α*, *IL-1*, *IL-6*, *IL-12*). CXC chemokines (*CXCL12*, *CXCL10*) and CC chemokines (*CCL3*, *CCL4*) production (57).

Studies have shown that chlorpyrifos induced immunosuppression in Nile tilapia, accompanied by decreased expression of *IL-1β*, *IL-8* and *IFN-γ* (58), embryonic exposure to PFOS induced immunosuppression in the fish larvae of marine medaka with up-regulated *IL-10* and down-regulated *IL-1β* (59).The results of this study were consistent with those above. Transcriptome cluster analysis showed that the majority of immune-related genes in the Myx group presented a down-regulated pattern compared with the control group, implying diminished immunity. Additionally, the Myx group showed a striking up-regulation of anti-inflammatory factor *IL-10* and a marked down-regulation of pro-inflammatory factors *IL-8* and *IL-1β*, possibly due to the up-regulation of *IL-10* inhibited the production of key first-line defense cytokines and chemokines, thereby inhibiting the recruitment to inflammatory sites and activation of immune cells. Zap-70 ^+^ staining additionally revealed that there was no discernible difference in T/NK cell signal in gills between Myx group and control group after parasite infection, which together with these findings demonstrated that *Myxobolus ampullicapsulatus* caused the goldfish’s immune system to be suppressed.

## Conclusion

In conclusion, this investigation demonstrated that, firstly, the *myxobolus* isolated from the gill of goldfish was identified as *Myxobolus ampullicapsulatus*. Secondly, the potential mechanisms of infecting goldfish gill with *Myxobolus ampullicapsulatus* may be apoptosis activation mediated by p53-Bcl2/Bax signaling pathway and immunosuppression.

## Data availability statement

The datasets presented in this study can be found in online repositories. The names of the repository/repositories and accession number(s) can be found below: Sequence Read Archive (SRA) at the NCBI repository, accession number: PRJNA865864.

## Ethics statement

The animal study was reviewed and approved by Animal Care and Use Committee of Sichuan Agricultural University.

## Authors contributions

XH, YG and JW designed the experiment and revised the manuscript. SL conducted most of the experiments, analyzed the data and wrote the manuscript. LL and FZ supported to do some of the experiments. DC and YO widely collected diseased healthy goldfish. SY, WL, and YW analyzed the results. All authors read and approved the final manuscript.

## Funding

This work was supported by Neijiang Normal University (NJTCSC09).

## Acknowledgments

We would like to thank Service Bio (Wuhan, Hubei province) for Zap-70 (99F2-Cell Signaling) Rabbit mAb antibody and TUNEL assay, and Magior Bio (Shanghai) for the transcriptome sequencing, as well as Lilai Bio (Chengdu, Sichuan province) for Transmission electron microscopy assay. In addition, we would like to thank “Key Laboratory of Sichuan Province for Fishes Conservation and Utilization in the Lpper Reaches of the Yangtze River, Neijiang Normal University” project for its support of this study.

## Conflict of interest

The authors declare that the research was conducted in the absence of any commercial or financial relationships that could be construed as a potential conflict of interest.

## Publisher’s note

All claims expressed in this article are solely those of the authors and do not necessarily represent those of their affiliated organizations, or those of the publisher, the editors and the reviewers. Any product that may be evaluated in this article, or claim that may be made by its manufacturer, is not guaranteed or endorsed by the publisher.
